# Two-stage treatment in patients with patients with high-energy femoral fractures does not lead to an increase in deep infectious complications: a propensity score analysis

**DOI:** 10.1007/s00068-017-0822-6

**Published:** 2017-07-28

**Authors:** S. A. Dingemans, M. A. T. Sier, R. W. Peters, J. C. Goslings, T. Schepers

**Affiliations:** Trauma Unit, Department of Surgery, Academic Medical Center, Meibergdreef 9, PO Box 22660, 1100 DD Amsterdam, The Netherlands

**Keywords:** Femoral fracture, Intramedullary nailing, External fixator, Propensity score analysis

## Abstract

**Purpose:**

In patients following severe trauma sometimes the physiological condition or soft tissue status may not allow definitive fixation of a femoral fracture. In these patients, an external fixator can be placed to temporarily stabilise the fracture, after which definitive fixation can be performed in a second procedure. The aim of this study was to compare the postoperative wound infection and union rates of patients treated with direct intramedullary nailing (IMN) and patients treated with the ‘two-stage treatment’.

**Methods:**

All patients with high-energy femoral fractures treated with IMN between 2000 and 2016 in a single Level 1 trauma centre were eligible. Electronic charts were reviewed for patient and surgical characteristics; furthermore, the development of complications was noted. A propensity score analysis was performed to assess the attributed risk of the external fixator on the development of postoperative wound infections.

**Results:**

A total of 149 patients were included in this study; 93 underwent direct IMN and 56 underwent the two-stage treatment. Patients who underwent two-stage treatment were more severely injured, reflected by lower EMV and higher ISS on admission. Patients in the two-stage treatment group had a significant higher risk of postoperative wound infections (OR: 4.698, 95% CI: 1.203–18.339) but not a higher risk on deep postoperative wound infections (OR 2.345, 95% CI: 0.439–12.540). Union rate did not differ between the two groups (94% vs 94% NS).

**Conclusions:**

The two-stage treatment is a safe treatment option in patients with a high-energy femoral fracture in terms of postoperative wound infections. Union rates are also comparable between the two treatment groups.

## Introduction

High-energy femoral fractures occur relatively more often in a younger patient population and are frequently located in the shaft of the femur [1]. After a fracture, early stabilisation of the bone is essential for the healing process and the prevention of complications [2]. Early fixation contributes to preservation of blood supply and early and safe mobilisation [3].

In general, there are three surgical methods to stabilise femoral shaft fractures (1) external fixation (mostly) through the placement of metal pins in the bones, connected outside the body by bars and (2) intramedullary nailing (IMN), and/or (3) plate-osteosynthesis.

Fractures of the femoral shaft are preferably treated with intramedullary nailing since this provides the strongest mechanical fixation and is considered to be the best treatment to realise early mobilisation.

In open fractures, the integrity of the skin can be damaged by either the fractured bone itself or by an external force [4]. Open fractures are always considered to be contaminated [5]; however, the severity of the contamination depends on the severity (grade) of open fracture [6]. In case of an open fracture, the two-stage treatment (i.e., initial stabilisation with external fixation and definitive treatment by internal fixation in a secondary procedure) is frequently preferred, to diminish the risk of postoperative wound infections [5]. Furthermore, after a high-energy trauma, patients may suffer from multiple injuries and can therefore be too critically ill to tolerate the surgical stress or the operative time necessary for intramedullary fixation [7]. In these patients, external fixation is an expedient and minimally invasive method of temporary fracture stabilisation [8]. External fixation of the fracture has to take place as soon as possible, preferably within 6 h after the injury. The time between the removal of the external fixator and the implantation of the internal fixator is variable as it depends on the condition of the patient. However, it has been shown that delay of the conversion to intramedullary nailing leads to an increased risk of postoperative wound infections [9]. Disadvantages of the two-stage treatment are that the patient has to undergo two procedures and that pin-track infections may occur. These pin-track infections may be a porte d’entrée for micro-organisms causing deep infections [8].

Therefore, the primary aim of this study was to assess the safety of the two-stage treatment in terms of infectious complications. Furthermore, we aimed to compare the union rates between direct intramedullary nailing and two-stage treatment.

## Methods

### Study design

A retrospective cohort study was performed using the local Trauma registry from a single academic Level 1 trauma centre in the Netherlands. Adult patients operated between January 2000 and March 2016 by the Traumasurgery department were eligible. Patients were included if they sustained (1) a high-energy fracture of the femur temporarily stabilised with an external fixator followed by intramedullary nailing or (2) a high-energy fracture of the femur treated with direct intramedullary nailing (defined as placement of an intramedullary device within 12 h following trauma).

Exclusion criteria were: (1) patients with a traumatic amputation through or above the knee and (2) patients treated with a different external fixator than bars and pins.

High-energy fractures were defined conform the ATLS^®^ guidelines and caused by; a motor vehicle-related traffic injury, firearm incident, fall from height >2 m, fall at high speed (e.g., fall with bicycle), aircraft accident, a crash with an object at high speed or a crash with a heavy object [10].

The decision to treat patients with direct intramedullary nailing or the two-stage treatment was made by the attending trauma surgeon based on his/her preference and the patient’s condition.

In case of an open fracture patients were treated with empiric antibiotics at the emergency department (i.e., penicillin combined with a beta-lactamase inhibitor). All patients received routine antibiotic prophylaxis (i.e., first generation cephalosporin) prior to surgical, in case of a Gustilo grade 3 open fracture gentamicin was added. Furthermore, intravenous therapy with a cephalosporin was continued for 3 days postoperatively in these patients.

### Outcome measures

Primary endpoint was postoperative wound infection defined by the criteria of the Centers for Disease Control and Prevention (CDC) [11]. Infections were subdivided into superficial wound infections and deep wound infection. Superficial infections were classified as infections requiring local wound treatment or oral antibiotics, deep infections were those requiring a surgical procedure or intravenous antibiotics. Only infections occurring after the placement of the external fixator or intramedullary nail, before operative removal of hardware and occurring within the time of follow-up were taken into consideration.

Secondary outcomes were; (mal-)union, leg shortening, compartment syndrome, and implant removal. Union was defined as weight-bearing ability occurring within the time of follow-up. Non-union was defined as a fracture that did not heal and needed an operative procedure for delayed union of the fracture. Mal-union was defined as union which required surgery due to significant deformity (e.g., varus, valgus or rotation). Leg shortening was defined as shortening of the femur which required an operational intervention.

### Data collection

The following data were collected from the patient charts: age, gender, American Society of Anaesthesiologists score (ASA), comorbidities, substance abuse(alcohol, nicotine and/or drugs), trauma mechanism, Glasgow Coma Scale (GCS) upon admittance, Injury Severity Score (ISS), the presence of an open fracture and Gustilo classification, time to external fixation/intramedullary nailing, time of surgery (in or outside working hours), length of stay at the intensive care unit (ICU), duration of hospitalisation, number of reoperations, time to weight-bearing, infectious complications (non-)union, mal-union, compartment syndrome and in-hospital mortality.

### Statistical analysis

Normality was assessed using histograms and normality tests. Baseline characteristics were checked for differences between the two groups. Chi-square, independent *t* tests and Mann–Whitney U tests were used where appropriate. Statistical analysis was performed using SPSS (v. 23.0 IBM, Chicago, Ill).

#### Propensity scoring

A propensity score was used to adjust for the risk of opting for the two-stage protocol. As mentioned before, the choice of therapy depends on the condition of the patient and wound. To ensure an equivalent comparison of the development of infectious complications following two-stage treatment or direct IMN, the patient groups have to be comparable in baseline patient characteristics which may influence the development of infections complications.

A propensity score is the probability of treatment assignment conditional on baseline characteristics. The propensity score allows one to design and analyse an observational (non-randomised) study in a way that it simulates some of the particular characteristics of a randomised controlled trial [12]. The covariate adjustment using propensity score method was used for this study. Using this approach, the outcome variable is regressed on an indicator variable denoting treatment status and the estimated propensity score. In this study, the outcome variable was infection and the indicator variable denotes the two-stage treatment or direct intramedullary nailing. This calculation requires two regressions. In the first regression, a propensity score for treatment is calculated, using treatment assignment as the outcome. Confounders must be included; important covariates can also be taken into account. Multivariable regression was used to detect confounders. The confounders included in this study were ISS and open fractures.

Subsequently, a dichotomous regression was performed to calculate the propensity score. Next, a new regression is performed to measure the probability of the development of an infection (our primary outcome) following definitive fixation. In this regression, the previously calculated propensity score and treatment were included. This calculation provides an outcome that is adjusted for the confounders, covariates and the difference in baseline characteristics between the patients treated with the two-stage treatment and patients treated with direct intramedullary nailing [13]. The effect of the two-stage treatment on the development of infectious complications using the propensity score is expressed in Odds Ratio’s (OR).

## Results

### Patients

A total of 152 patients were eligible for inclusion. Three patients were excluded, reasons for exclusion were: amputation through or above the knee (*n* = 2) and traction splint instead of external fixation by rods and bars (*n* = 1). A total of 149 patients were included in the study cohort (*n* = 56 two-stage treatment group, *n* = 93 direct intramedullary nailing group). Patients were predominantly male (80%) with a median age of 30 years. Mechanisms of injury included motor vehicle-related traffic injury (*n* = 104), fall from height (*n* = 27), crash with object (*n* = 5), firearm incident (*n* = 5), fall at high speed (*n* = 6) and airplane crash (*n* = 1). The majority of the patients had an admission ASA of 1, median ISS was 13 and a median EMV 15. Open fractures occurred in 22% of the patients. Time to initial treatment did not differ between the two groups. Baseline characteristics are shown in Table 1.

**Table 1:** Patient characteristics

	Two-stage treatment (*n* = 56)	Direct intramedullary nailing (*n* = 93)	*p* value
Age (years)*	26.5 [18.25–42]	33 [23.5–41]	0.02^a^
Male gender (%)	41 (73)	78 (84)	0.12^b^
ASA score (%)			0.28^b^
1	18 (56)	47 (61)	
2	4 (13)	18 (23)	
3	4 (13)	7 (9)	
4	5 (16)	4 (5)	
5	1 (1)	1 (1)	
Unknown	24	16	
Diabetes mellitus (%)	2 (4)	2 (2)	0.63^b^
Mechanism of injury (%)			0.10^b^
Motor vehicle-related traffic injury	45 (80)	59 (64)	
Firearm incident	1 (2)	4 (4)	
Fall from height	7 (13)	20 (22)	
Fall at high speed	1 (2)	5 (6)	
Crash with an object at high speed or of heavy weight	2 (2)	4 (4)	
Unknown	0	1	
EMV^*^	15 [8–15]	15 [15–15]	<0.001^a^
ISS score^*^	22 [13–28.5]	10 [9–18.5]	<0.001^a^
Open fracture (%)	18 (32%)	15 (16%)	0.02^b^
Gustilo classification (%)			0.08^b^
0	38 (78)	78 (89)	
1	5 (10)	5 (6)	
2	3 (6)	5 (6)	
3	3 (6)	0	
Unknown	7	5	

^*^ Variables are denoted as median [IQR]

^a^ Mann–Whitney *U* test

^b^ Pearson *χ* ^2^ test

### Direct intramedullary nailing vs Two-stage treatment

In general, patients treated with two-stage treatment were younger. Patients undergoing two-stage treatment were more severely injured, which is reflected by significant differences in the EMV scores, ISS and higher rate of open fractures. Median time between placement of an external fixator and conversion to internal fixation was 5 days (IQR 3–7.75). Treatment characteristics are available in Table 2. The ICU length of stay was longer in the group of patients treated with the two-stage treatment, as well as a total length of hospitalisation.

**Table 2:** Treatment characteristics

	Two-stage treatment (*n* = 56)	Direct intramedullary nailing (*n* = 93)	*p* value
Operated outside office hours (%)	42 (78)	65 (70)	0.30^b^
Time to initial treatment^*^ (days) [IQR]	0 [0–0]	0 [0–0]	0.19^a^
Reaming	50 (89)	80 (86)	0.29^b^

^*^ Variables are denoted as median [interquartile range]

^a^ Mann–Whitney *U* test

^b^ Pearson *χ* ^2^ test

### Follow-up characteristics

The median time of follow-up of all patients was 19 months; the median duration of follow-up in the two-stage treatment group was 22.5 months (IQR 10.25–37) and 16 months (IQR 2–37.5) in the intramedullary nailing group. Twelve patients were lost to follow-up within 1 month; the majority underwent direct intramedullary nailing (11/12). These patients had low ISS, high EMV scores and were less likely to incur a non-systemic complication.

### Complications

Table 3 shows the incidence of the recorded complications. The most frequent complications were postoperative wound infection and non-union. No significant differences were observed between the two groups; however, there was a trend towards a higher number of infectious complications in the two-stage treatment group. In 15 (27%) of the patients in the two-stage group and in 20 (22%) of the patients in the direct IMN group a reoperation was required, this difference was not statistically significantly (*p* = 0.46). Median number of reoperations did also not differ statistically significantly between the two groups (0 [IQR 0–1] vs 0 [IQR: 0–1] [*p* = 0.87]). Four fatal outcomes were observed, two in each group (*p* = 0.63).

**Table 3:** Complications

	Two-stage treatment (*n* = 56)	Direct intramedullary nailing (*n* = 93)	*p* value
Non-systemic complication (%)	17 (30)	21 (23)	0.26^b^
Missing	1	0	
Infection (%)	8 (14)	4 (4)	0.06^c^
Superficial (%)	4 (7)	1 (1)	0.07^c^
Deep (%)	4 (7)	3 (3)	0.43^c^
Non-union (%)	11 (20)	13 (14)	0.34^b^
Mal-union (%)	1 (2)	2 (2)	0.99^c^
Leg shortage	0 (0)	2 (2)	0.53^c^
Compartment syndrome (%)	3 (5)	2 (2)	0.37^c^
Systemic complication (%)			0.87^b^
ARDS	2 (4)	0 (0)	
Multi organ failure	1 (2)	1 (1)	
Sepsis	1 (2)	3 (3)	
Shock	2 (4)	1 (1)	
Rhabdomyolysis	3 (5)	3 (3)	
Fat-embolism syndrome	1 (2)	1 (1)	
Critical illness polyneuropathy	1 (2)	1 (1)	
In-hospital mortality	2 (4)	2 (2)	0.63^c^

^a^ Mann–Whitney *U* test

^b^ Pearson *χ* ^2^ test

^c^ Fishers exact test

### Predictive factors for complications

We examined the safety of the two-stage treatment in patients with energy femoral fractures, exploring the influence on the development of infections of the two-stage treatment itself. Overall, a trend towards higher infection rate in the two-stage treatment was observed (*p* = 0.06) (Table 3).

### Propensity score analysis

Using the covariate adjustment method, a propensity score analysis was performed on the development of postoperative wound infections following surgical fixation of a femur fracture. A significant relation was found between the two-stage treatment and the development of any infectious complication (OR 4.698, 95% CI: 1.203–18.339). Subsequently, a new logistic regression, using the same propensity score, was performed to assess the correlation between the two-stage treatment and superficial and deep wound infections. A statistically significant relation between the development of superficial wound infections and the use of two-stage treatment was found (OR 11.588 95% CI: 1.116–120.345); however, no significant relationship between the development of deep wound infections and the use of the two-stage treatment was found (OR 2.345, 95% CI: 0.439–12.540).

### Fracture healing

Union was observed in 94% of the patients in both the two-stage treatment group and the direct intramedullary nailing group. Median time to weight-bearing was significantly shorter in the direct IMN group (98 days vs 75 days *p* = 0.007); median time to radiographic union was not statistically significantly different between the two groups (13 months vs 17 months *p* = 0.44). The rate of implant removal did not differ between the two groups (43% vs 43% *p* = 0.99).

## Discussion

We observed an increased risk of postoperative wound infections in patients treated with the two-stage treatment in comparison with patients undergoing direct IMN. However, regarding the development of deep postoperative wound infections, no significant difference between the two groups was found. Furthermore, no correlation between the two-stage treatment and non-union was found.

Patients treated according to the two-stage treatment were more severely injured compared to patients treated with direct intramedullary nailing. This is reflected by the ISS which was significantly higher in the two-stage treatment group. The ISS of our study groups were equal, if not lower, to the ISS reported in the currently available studies on this patient population [8, 14, 15]. The difference in ISS between the two groups is, however, similar to previous studies. Moreover, higher EMV scores were observed in our study population when compared with the literature [8, 14, 15]. The lower ISS and higher EMV scores might be attributable to the fact that we included all patients with a high-energy trauma, which does not necessarily mean that they sustained multiple injuries. Most studies on this subject only included multiple injured patients or patients with high ISS [8, 15]. An open fracture was more often seen in the group with the two-stage treatment.

The infection rate in this study was higher than the infection rates found in the literature [8, 16–18]. However, the rate of deep infections corresponds with previous studies [7, 16]. The increased amount of wound infections may be attributable to improved registration of infections, in specific superficial wound infections in our hospital. In the Netherlands, all complications have to be reported to the national health care institute. As a result of this, complications are reviewed in our hospital twice every week for each discharged patient. This leads to an exact registration of all complications (including superficial wound infections). We have reviewed patient charts and discharge letters which have proven to extract all complications [19].

After adjustment for the covariates using propensity score, the two-stage treatment correlated significantly with the development of infections; however, this difference disappeared when we assessed the correlation with the development of deep wound infections. These results imply a relation between two-stage treatment and the development infections in general, but not the risk of developing a deep wound infection. Possibly external fixators are a porte d’entree for micro-organism the colonise the wound, however the bacterial inocula is probably too small to cause deep infections [9]. However, antibiotic prophylaxis and perhaps debridement of pin tracks are essential as Clasper et al. showed in an animal model that in specimens with a pin-track infection, bony infection was widespread [20]. These results show that physicians should be wary of infection complications and antibiotic therapy may be considered at a low threshold.

Our union rates are comparable or even higher to those reported in the literature [21]. Furthermore, we observed no significant difference in union rates between the two-stage treatment group and direct IMN group. We did not expect such difference to exist; however, it does strengthen our belief that the staged treatment is a viable option in patients with high-energy femoral fractures.

Timing of the internal fixation has shown to be of importance. In a paper by Morshed et al., it was shown that delayed intramedullary fixation reduced mortality, especially in the critically ill. [22] Exchange nailing has to be performed as soon as the condition of the patients allows definitive surgical fixation as patients can start rehabilitation from this point. Time to definitive fixation in our study is comparable to earlier reports [7, 23].

The high rates of loss to follow-up may be explained by the overrepresentation of foreign patients who are transferred to their native country following initial treatment and patients under police investigation who are transferred to a prison ward once in a stable condition.

For this study, some limitations have to be considered. First, there is no universal definition of a high-energy trauma. The difference between high- and low-energy trauma was based on literature and at the discretion of the investigators [10, 18]. Some of the patient charts lacked a detailed description of the mechanism of injury, which impeded the estimation of the energetic force. Second, the covariate adjustment using the propensity score method is considered to be less reliable than the propensity score matching methodology. Matching was sadly not possible due to the small amount of patients. Using covariate adjustment method, the results may be subject to bias. To prevent this, we excluded missing data and included confounders in the calculation. Therefore, the bias is considered to be minimal [24]. However, this resulted in the fact that the ASA score could not be included in the propensity score regression due to missing data. This is a limitation since the ASA score is known to be influential on the development of infections [25]. On the other hand, both the open fractures and the ISS score have been shown to be influential on the development of complications and were included in the propensity score [9, 15]. The present study was also limited by a small sample size. This is, however, due to the uncommon nature of this injury. In comparison with previous studies, this study presents one of the largest cohorts yet. Lastly, no laboratory variables were included in this study. The absence of these variates is a limitation considering the potential predictive value of lactate and the APACHE II score for the development of complications (e.g., compartment syndrome and mortality) [26, 27].

To our knowledge, this is the first study comparing the development of infection using a propensity score analysis, to investigate the risk of developing a postoperative wound infection exclusively based on choice of initial treatment.

## Conclusion

Patients treated with the two-stage treatment are at risk for the development of postoperative wound infections following definitive treatment; there is, however, no increased risk of developing deep wound infections. Two-stage treatment is a safe treatment for patients suffering from a high-energy femoral fracture. Furthermore, high union rates are achieved in both patients treated with the two-stage treatment as well as in patients treated with direct intramedullary nailing.

## References

[1] Shane E, Burr D, Ebeling PR, Abrahamsen B, Adler RA, Brown TD (2010). Atypical subtrochanteric and diaphyseal femoral fractures: report of a task force of the American society for bone and mineral research. J Bone Miner Res [Internet]..

[2] Bone LB, Johnson KD, Weigelt J, Scheinberg R (1989). Early versus delayed stabilization of femoral fractures. A prospective randomized study. J Bone Joint Surg Am..

[3] Rüedi TP, Buckley R, Moran CG (2009). AO principles of fracture management. Ann R Coll Surg Engl..

[4] Wyatt JP, Illingworth RN, Graham CA, Hogg K (2012). Oxford handbook of emergency medicine.

[5] Gustilo RB, Anderson JT (1976). Prevention of infection in the treatment of 1025 open fractures of long bones: retrospective and prospective analyses. J Bone Joint Surg Am..

[6] Templeman DC, Gulli B, Tsukayama DT, Gustilo RB. Update on the management of open fractures of the tibial shaft. Clin Orthop Relat Res. 1998;(350):18–25. 9602796

[7] Scalea TM, Boswell SA, Scott JD, Mitchell KA, Kramer ME, Pollak AN (2000). External fixation as a bridge to intramedullary nailing for patients with multiple injuries and with femur fractures: damage control orthopedics. J Trauma..

[8] Nowotarski PJ, Turen CH, Brumback RJ, Scarboro JM (2000). Conversion of external fixation to intramedullary nailing for fractures of the shaft of the femur in multiply injured patients. J Bone Joint Surg Am..

[9] Harwood PJ, Giannoudis PV, Probst C, Krettek C, Pape H-C (2006). The risk of local infective complications after damage control procedures for femoral shaft fracture. J Orthop Trauma..

[10] Wordsworth M, Lawton G, Nathwani D, Pearse M, Naique S, Dodds A (2016). Improving the care of patients with severe open fractures of the tibia: the effect of the introduction of major trauma networks and national guidelines. Bone Joint J..

[11] Mangram AJ, Horan TC, Pearson ML, Silver LC, Jarvis WR (1999). Guideline for prevention of surgical site infection, 1999. Hospital infection control practices advisory committee. Infect Control Hosp Epidemiol..

[12] Olthof DC, Joosse P, Bossuyt PMM, de Rooij PP, Leenen LPH, Wendt KW (2016). Observation versus embolization in patients with blunt splenic injury after trauma: a propensity score analysis. World J Surg..

[13] Austin PC (2011). An introduction to propensity score methods for reducing the effects of confounding in observational studies. Multivariate Behav Res..

[14] Rixen D, Steinhausen E, Sauerland S, Lefering R, Maegele MG, Bouillon B (2016). Randomized, controlled, two-arm, interventional, multicenter study on risk-adapted damage control orthopedic surgery of femur shaft fractures in multiple-trauma patients. Trials..

[15] Pape H-C, Rixen D, Morley J, Husebye EE, Mueller M, Dumont C (2007). Impact of the method of initial stabilization for femoral shaft fractures in patients with multiple injuries at risk for complications (borderline patients). Ann Surg..

[16] Pape H-C, Hildebrand F, Pertschy S, Zelle B, Garapati R, Grimme K (2002). Changes in the management of femoral shaft fractures in polytrauma patients: from early total care to damage control orthopedic surgery. J Trauma..

[17] Höntzsch D, Weller S, Engels C, Kaiserauer S (1993). Change in the procedure from external fixator to intramedullary nailing osteosynthesis of the femur and tibia. Aktuelle Traumatol..

[18] Wolinsky PR, McCarty E, Shyr Y, Johnson K (1999). Reamed intramedullary nailing of the femur: 551 cases. J Trauma..

[19] Ubbink DT, Visser A, Gouma DJ, Goslings JC. Registration of surgical adverse outcomes: a reliability study in a university hospital. BMJ Open. 2012;2(3). doi:10.1136/bmjopen-2012-000891 10.1136/bmjopen-2012-000891 PMC3367156 22637372

[20] Clasper JC, Stapley SA, Bowley DM, Kenward CE, Taylor V, Watkins PE (2001). Spread of infection, in an animal model, after intramedullary nailing of an infected external fixator pin track. J Orthop Res..

[21] Li A-B, Zhang W-J, Guo W-J, Wang X-H, Jin H-M, Zhao Y-M (2016). Reamed versus unreamed intramedullary nailing for the treatment of femoral fractures: a meta-analysis of prospective randomized controlled trials. Medicine (Baltimore)..

[22] Morshed S, Miclau T, Bembom O, Cohen M, Knudson MM, Colford JM (2009). Delayed internal fixation of femoral shaft fracture reduces mortality among patients with multisystem trauma. J Bone Joint Surg Am..

[23] Scannell BP, Waldrop NE, Sasser HC, Sing RF, Bosse MJ (2010). Skeletal traction versus external fixation in the initial temporization of femoral shaft fractures in severely injured patients. J Trauma..

[24] Lonjon G, Porcher R, Ergina P, Fouet M, Boutron I (2016). Potential pitfalls of reporting and bias in observational studies with propensity score analysis assessing a surgical procedure: a methodological systematic review. Ann Surg..

[25] Ridgeway S, Wilson J, Charlet A, Kafatos G, Pearson A, Coello R (2005). Infection of the surgical site after arthroplasty of the hip. J Bone Joint Surg Br..

[26] Kosir R, Moore FA, Selby JH, Cocanour CS, Kozar RA, Gonzalez EA (2007). Acute lower extremity compartment syndrome (ALECS) screening protocol in critically ill trauma patients. J Trauma..

[27] Knaus WA, Wagner DP, Draper EA, Zimmerman JE, Bergner M, Bastos PG (1991). The APACHE III prognostic system. Risk prediction of hospital mortality for critically ill hospitalized adults. Chest..

